# Diaphragm muscle sarcopenia into very old age in mice

**DOI:** 10.14814/phy2.14305

**Published:** 2020-01-06

**Authors:** Pangdra Vang, Amrit Vasdev, Wen‐Zhi Zhan, Heather M. Gransee, Gary C. Sieck, Carlos B. Mantilla

**Affiliations:** ^1^ Department of Physiology & Biomedical Engineering Mayo Clinic Rochester Minnesota; ^2^ Department of Anesthesiology & Perioperative Medicine Mayo Clinic Rochester Minnesota

**Keywords:** aging, cross‐sectional area, transdiaphragmatic pressure, ventilation

## Abstract

Sarcopenia is the age‐related decline of skeletal muscle mass and function. Diaphragm muscle (DIAm) sarcopenia may contribute to respiratory complications, a common cause of morbidity and mortality in the elderly. From 6 to 24 months (mo) of age, representing ~100% and ~80% survival in C57BL/6 × 129 male and female mice, there is a significant reduction in DIAm force generation (~30%) and cross‐sectional area (CSA) of type IIx and/or IIb muscle fibers (~30%), impacting the ability to perform high force, non‐ventilatory behaviors. To date, there is little information available regarding DIAm sarcopenia in very old age groups. The present study examined DIAm sarcopenia in C57BL/6 × 129 male and female mice at 24, 27, and 30 mo, representing ~80%, ~60%, and ~30% survival, respectively. We hypothesized that survival into older ages will show no further worsening of DIAm sarcopenia and functional impairment in 30 mo mice compared to 24 or 27 mo C57BL/6 × 129 mice. Measurements included resting ventilation, transdiaphragmatic pressure (Pdi) generation across a range of motor behaviors, muscle fiber CSA, and proportion of type‐identified DIAm fibers. Maximum Pdi and resting ventilation did not change into very old age (from 24 to 30 mo). Type IIx and/or IIb fiber CSA and proportions did not change into very old age. The results of the study support a critical threshold for the reduction in DIAm force and Pdi such that survival into very old age is not associated with evidence of progression of DIAm sarcopenia or impairment in ventilation.

## Graphical abstract

The purpose of this study was to examine DIAm sarcopenia into very old age in C57BL/6 × 129 male and female mice during a period (24–30 mo) of substantial reduction in survivorship (~80% vs. ~30%, respectively). The results of the study support a critical threshold for the reduction in DIAm force and Pdi such that survival into very old age is not associated with evidence of progression of DIAm sarcopenia or impairment in ventilation.

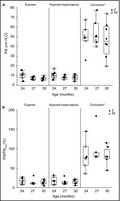

## 1 INTRODUCTION

Respiratory complications and diseases are common causes of morbidity and mortality in the elderly (Heron, 2017; Yang et al., 2017). Sarcopenia encompasses the age‐related decline of skeletal muscle mass and/or function, and is particularly evident in respiratory muscles including the diaphragm (DIAm) (Cesari et al., 2012; Elliott, Greising, Mantilla, & Sieck, 2016; Fielding et al., 2011; Greising, Mantilla, Gorman, Ermilov, & Sieck, 2013; Greising, Mantilla, Medina‐Martinez, Stowe, & Sieck, 2015). The DIAm has a heterogeneous composition of motor units, which are recruited to accomplish ventilation and a range of life‐sustaining motor behaviors important for airway clearance (e.g., sneezing or coughing) (Enad, Fournier, & Sieck, 1989; Fournier & Sieck, 1988; Sieck & Fournier, 1989; Sieck, Fournier, & Enad, 1989). Transdiaphragmatic pressure (Pdi) measurements are used clinically and experimentally across species to determine the forces generated by the DIAm (Bazzy & Haddad, 1984; Gill, Mantilla, & Sieck, 2015; Greising, Sieck, Sieck, & Mantilla, 2013; Mantilla, Seven, Zhan, & Sieck, 2010; Sieck, 1991; Sieck & Fournier, 1989). Previous studies show a ~30% reduction in Pdi_max_ (generated by maximal activation of the DIAm via bilateral phrenic nerve stimulation) with age (6–24 months (mo) (Elliott, Greising, et al., 2016; Greising, Medina‐Martínez, Vasdev, Sieck, & Mantilla, 2015; Khurram et al., 2018).

The mechanisms responsible for sarcopenia of respiratory muscles including the DIAm, and the associated loss of muscle mass or force, are presently unknown. Previous studies suggest that DIAm sarcopenia is associated with a loss of motor neurons and fiber type‐selective atrophy (Greising, Mantilla, et al., 2015; Greising, Medina‐Martínez, et al., 2015; Khurram et al., 2018; Mantilla & Sieck, 2011a), dysfunction at the neuromuscular junction (Fogarty, Gonzalez Porras, Mantilla, & Sieck, 2019; Greising, Stowe, Sieck, & Mantilla, 2015; Holter, Kierulf, & Refsum, 1987; Mantilla & Sieck, 2011a), or intrinsic muscle changes including mitochondrial degeneration and autophagy (Gonzalez Porras, 2018). For instance, from 6 to 24 mo in C57BL/6 male and female mice, there is fiber type‐selective atrophy of large, high force generating type IIx and/or IIb fibers (Elliott, Omar, Mantilla, & Sieck, 2016; Greising, Mantilla, et al., 2013, 2015; Greising, Medina‐Martínez, et al., 2015; Greising, Vasdev, Zhan, Sieck, & Mantilla, 2017). However, a recent study found significant DIAm hypertrophy of type IIx fibers (~30% increase in fiber cross‐sectional area (CSA)) in female CD‐1 mice at 20 mo compared to 5 mo (Messa et al., 2019). Mouse lifespan decreases with age, with ~100% survivorship at 6 mo, ~80% at 24 mo, ~60% at 27 mo, and ~30% survival at 30 mo in male and female C57BL/6 mice (Elliott, Mantilla, Pabelick, Roden, & Sieck, 2016; Flurkey, 2009; Greising, Mantilla, et al., 2013; Turturro et al., 1999), compared to ~50% in 20 mo female CD‐1 mice (Messa et al., 2019; Navarro, Sanchez Del Pino, Gomez, Peralta, & Boveris, 2002). To date, few studies have examined changes in DIAm morphology and function associated with survivorship into very old age (<50% survival). Furthermore, it is presently unknown if fiber type‐selective changes in DIAm CSA continue into advanced age and contribute to functional decline. Given the DIAm performs life‐sustaining motor behaviors and progressive loss‐of‐function would be incompatible with life, we hypothesized that survival into older ages will show no further worsening of DIAm sarcopenia and functional impairment in 30 mo mice compared to 24 or 27 mo C57BL/6 × 129 mice.

## 2 MATERIALS AND METHODS

All protocols were approved by the Institutional Animal Care and Use Committee at Mayo Clinic and were compliant with the American Physiological Society and National Institute of Health Guidelines. All experiments were designed according to the principles of animal use for gerontological research (Miller & Nadon, 2000).

### 2.1 Animals

C57BL/6 × 129 male and female mice were bred and naturally aged in a pathogen‐free colony maintained at the Mayo Clinic. Mice were group‐housed by sex up to five per cage and maintained on a 12 hr light‐dark schedule with ad libitum access to food (PicoLab Rodent Diet 20; LabDiet, St. Louis, MO) and water. Whole body plethysmography was evaluated in 24 mo (*n* = 5 male, *n* = 5 female), 27 mo (*n* = 5 male, *n* = 5 female), and 30 mo (*n* = 5 male, *n* = 5 female) C57BL/6 × 129 mice. Pdi generation was measured in 24 mo (*n* = 4 male, *n* = 3 female), 27 mo (*n* = 2 male, *n* = 5 female), and 30 mo (*n* = 2 male, *n* = 6 female) C57BL/6 × 129 mice. Survival estimates are based on our C57BL/6 × 129 colony and are in agreement with published data (Elliott, Mantilla, et al., 2016; Flurkey, 2009; Greising, Mantilla, et al., 2013; Turturro et al., 1999; Yuan et al.., 2009).

### 2.2 Whole body plethysmography

Ventilatory parameters were measured in awake mice during eupnea (spontaneous breathing of room air) using a whole body plethysmography system (Buxco Inc.). Before all recordings, the system was calibrated according to the manufacturer's recommendations. Recordings were taken after 10 min of acclimatization and recorded for one hour. Recorded parameters included tidal volume (V_T_), minute ventilation (VE), ventilatory frequency, inspiratory and expiratory durations, and inspiratory duty cycle (time of inspiration as fraction of inter‐breath period).

### 2.3 Transdiaphragmatic pressure measurements

Male and female C57BL/6 × 129 mice were anesthetized by an intraperitoneal injection of fentanyl (0.3 mg/kg), droperidol (15 mg/kg), and diazepam (5 mg/kg). Anesthetized mice were placed on a heating pad and monitored continually for temperature (ThermoCouple TMD‐50; Amprobe), oxygen saturation, and heart rate (MouseOx pulse oximeter; STARR LifeSciences). Palpebral reflex and pain response were monitored to ensure depth of anesthesia. Pdi measurement has been described previously (Greising, Mantilla, et al., 2015; Greising, Mantilla, & Sieck, 2016; Greising, Sieck, et al., 2013; Khurram et al., 2018; Khurram, Sieck, & Mantilla, 2017). Briefly, a tracheostomy was performed and the trachea was cannulated (19G) (Greising, Mantilla, et al., 2013). Spontaneous ventilation was maintained, the abdomen was bound, and Pdi was recorded using two 3.5 French Millar solid‐state pressure catheters (SPR‐524; Millar Instruments). The catheters were inserted through the mouth and positioned in the esophagus and stomach. The placement of catheters was confirmed by positive deflection of the stomach signal and negative deflection of the esophageal signal across behaviors. If placed incorrectly, catheters were adjusted. The tension of the abdominal binding was adjusted until optimal gastric and esophageal pressures were achieved during eupnea, thus approximating isometric conditions. For both esophageal and stomach signals during stimulation, it was verified that there were no movement artifacts, and the duration and stimulation period of the Pdi response were the same.

Recordings of Pdi across behaviors were obtained in order, during: (a) spontaneous breathing of room air (eupnea) for 5 min, (b) spontaneous breathing challenged by exposure to hypoxia (10% O_2_)‐hypercapnia (5% CO_2_) for 5 min, (c) spontaneous breathing against tracheal occlusion for 15 s, and (d) responses induced by bilateral phrenic nerve stimulation (Pdi_max_; 0.5 ms‐duration pulses at 150 Hz in 300‐ms trains repeated each second) using straight bipolar electrodes (FHC).

The pressure data were collected using a PowerLab 8/35 data‐acquisition system and analyzed using LabChart (ADInstruments). Pdi measurements in LabChart were sampled at 100 Hz and band‐pass filtered (0.3–30 Hz). The data was exported into MATLAB (MathWorks) for analysis using a custom‐designed semi‐automated program to detect baseline and peak amplitude. In addition, the tension‐time index was used as an estimate of the efficiency of DIAm activation and was calculated as Pdi amplitude (normalized to Pdi_max_)*Duty Cycle (Bellemare & Grassino, 1982).

### 2.4 DIAm histomorphological analysis

At the terminal experiment, male and female C57BL/6 × 129 mice were anesthetized by an intraperitoneal injection of fentanyl (0.3 mg/kg), droperidol (15 mg/kg), and diazepam (5 mg/kg) and then euthanized by exsanguination. DIAm were dissected, frozen, and stored at −80°C until further examination. Muscle sections were cut into 10 μm thick cross‐sections for histological classification, as in previous studies (Greising, Mantilla, et al., 2013, 2015; Greising, Medina‐Martínez, et al., 2015). Gross histologic examination of DIAm sections was performed on hematoxylin and eosin‐stained sections imaged with brightfield microscopy (BX50WI, Olympus). Additionally, muscle cross‐sections were histologically classified based on MyHC isoforms. Each section was labeled using primary antibodies for anti‐MyHC_Slow_ (BA‐F8; Developmental Studies Hybridoma Bank), anti‐MyHC_2A_ (SC‐71; Developmental Studies Hybridoma Bank), and laminin (L9393; Sigma) to visualize the sarcolemma. Cross‐sections were treated with appropriate fluorescently‐conjugated secondary antibodies. Muscle fibers were classified as type I, type IIa, and type IIx and/or IIb based on the expression of MyHC_Slow_, MyHC_2A_, and the absence of staining, respectively (Elliott, Greising, et al., 2016; Greising, Mantilla, et al., 2013; Greising, Medina‐Martínez, et al., 2015).

Imaging of each DIAm section was obtained via confocal microscopy using an Olympus FluoView 1200 laser scanning confocal microscope system equipped with Argon (488 nm) and solid‐state (543 nm and 643 nm) lasers. Images were acquired in a 1,024 × 1,024 array with pixel dimensions (0.621 × 0.621 µm). Using Fluoview software, all confocal images were stored as 24‐bit multi‐TIFF files. Every confocal image was separated into three individuals colors (channels) in Meta Morph (Molecular Devices), saved as three 8‐bit TIFF files, and analyzed using the threshold, binarize and morphometric analysis functions in MetaMorph.

### 2.5 Statistics

JMP (SAS Institute Inc.) was used to analyze all data. Data for body mass were analyzed using a mixed linear model, with age, sex, and their interactions as fixed effects and animal as a random effect. Ventilatory parameters were analyzed across age with a one‐way ANOVA. Absolute and normalized Pdi amplitude were analyzed using a mixed linear model with age, behavior, and their interactions as fixed effects and animal as a random effect. DIAm CSA and fiber type‐specific relative contribution was analyzed using a mixed linear model with age, fiber type, and their interactions as fixed effects and animal as a random effect. DIAm fiber type proportions were analyzed by a two‐way ANOVA with age and fiber type as grouping variables. Tukey‐Kramer HSD *post hoc* analyses were conducted when appropriate. Unless otherwise specified, all data reported as the mean ± standard deviation of the mean (*SD*). Significance was accepted at *α* < .05 level.

## 3 RESULTS

### 3.1 Animal parameters

Naturally aged C57BL/6 × 129 mice (five male and five female in each age group) were studied at 24, 27, and 30 mo. The mean body mass was 27.2 ± 5.2 g and 32.3 ± 5.1 g for female and male mice, respectively, with an effect on body mass of sex (*F*_1,24_ = 7.7, *p* = .01), but no effect of very old age (*F*_2,24_ = 2.3, *p* = .12) or an age × sex interaction (*F*_2,24_ = 0.09, *p* = .91) (Table 1). In our colony of ~220 female and male mice, survival rates were 100% at 6 mo, 81% at 24 mo, 60% at 27 mo, and 33% at 30 mo.

**Table 1:** Body mass in C57BL/6 × 129 mice of very old age

Age	Female (g)^a^	Male (g)
24 (mo)	29.4 ± 6.3	35.2 ± 6.1
27 (mo)	26.8 ± 2.1	32.4 ± 3.0
30 (mo)	25.4 ± 6.3	29.4 ± 5.0

Note

Data analyzed by a mixed linear model with animal as a random effect (age × sex × animal) at 24, 27, and 30 months of age (mo; *n* = 5 for males and females in each age group); main effect of sex (*p* = .01), no effect of age (*p* = .12), or their interaction (*p* = .91). Data shown as mean ± *SD*.

a Female mice body mass (g) significantly different than male body mass (g).

### 3.2 Whole body plethysmography

Whole‐body plethysmography was successfully measured in all animals, and used to determine respiratory rate (RR), tidal volume (V_T_, normalized to body mass), minute ventilation (VE, normalized to body mass), and duty cycle in awake, unrestrained male and female C57BL/6 × 129 mice at 24, 27, and 30 mo (*n* = 10 for each age group) (Table 2). There was no effect on RR of very old age (*F*_2,27_ = 2.5, *p* = .10), average V_T_ (*F*_2,27_ = 1.1, *p* = .33), normalized VE (*F*_2,27_ = 0.68, *p* = .52), or duty cycle (*F*_2,27_ = 1.7, *p* = .20). Overall, the average RR across all (24–30 mo) age groups was 194.64 ± 52.8 min^‐1^, V_T_ was 0.009 ± 0.002 ml/g, VE was 1.74 ± 0.42 ml g^−1^ min^−1^, and duty cycle was 32% ± 7%.

**Table 2:** Ventilatory parameters measured in awake C57BL/6 × 129 mice using whole body plethysmography

Age	Respiratory rate (min^−1^)	Tidal volume (ml/g)	Minute ventilation (ml/g/min^−1^)	Duty cycle (%)
24 (mo)	216 ± 43	0.009 ± 0.002	1.8 ± 0.6	35 ± 9
27 (mo)	167 ± 32	0.010 ± 0.001	1.6 ± 0.3	31 ± 5
30 (mo)	202 ± 68	0.010 ± 0.003	1.8 ± 0.4	30 ± 5

Note

Data analyzed by one‐way ANOVA at 24, 27, and 30 months of age (mo; *n* = 10 for each age group). No age effect was evident on respiratory rate (*p* = .10), normalized tidal volume (*p* = .33), normalized minute ventilation (*p* = .52), or duty cycle (*p* = .20). Data shown as mean ± *SD*.

### 3.3 Transdiaphragmatic pressure measurement

**Figure 1:**
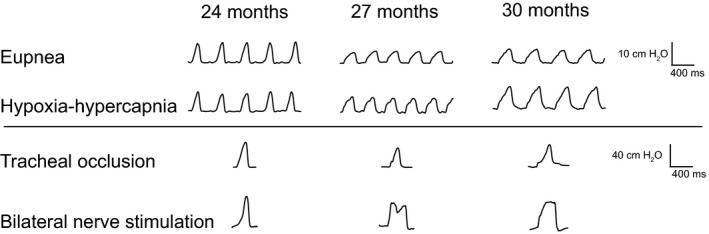
Representative transdiaphragmatic pressure (Pdi) tracings during various motor behaviors in 24, 27, and 30 month old C57BL/6 × 129 mice. Increasing Pdi is evident from eupnea and hypoxia‐hypercapnia to tracheal occlusion and bilateral nerve stimulation (Pdi_max_). Note bar represents 10 cm H_2_O for eupnea and hypoxia‐hypercapnia and 40 cm H_2_O for tracheal occlusion and bilateral nerve stimulation

**Figure 2:**
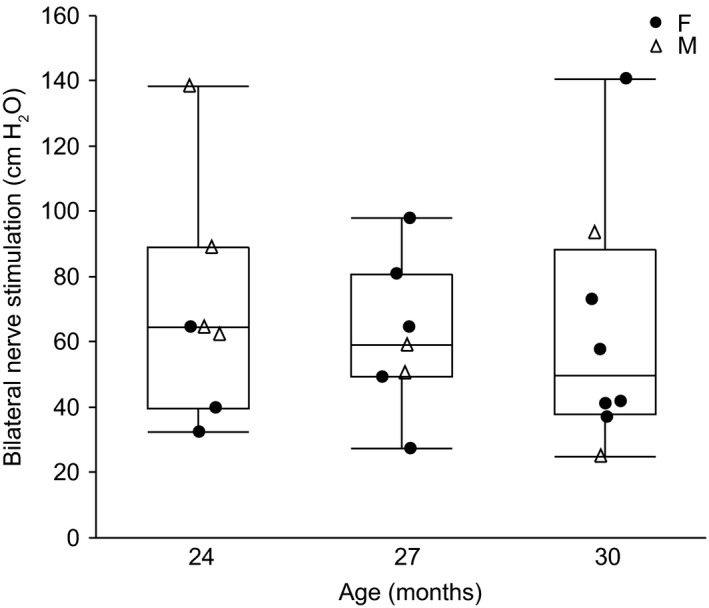
Maximal transdiaphragmatic pressure (Pdi_max_) generated during bilateral phrenic nerve stimulation in 24 (*n* = 7), 27 (*n* = 7), and 30 (*n* = 8) month old female (F; closed circle) and male (M; open triangle) C57BL/6 × 129 mice. Data points are the average Pdi_max_ amplitude from each animal. Boxplot represents median, 25th and 75th percentile and whiskers 10th and 90th percentiles. Data were analyzed by a mixed linear model with animal as a random effect (age × sex × animal); no effect of age (*p* = .92), sex (*p* = .56), or interaction (*p* = .92)

Pdi was successfully measured during eupnea, hypoxia‐hypercapnia, and tracheal occlusion in 24 mo (*n* = 7), 27 mo (*n* = 7), and 30 mo (*n* = 8) male and female C57BL/6 × 129 mice. Representative Pdi amplitude tracings from 24, 27, and 30 mo mice are shown in Figure 1. Regular breathing patterns were observed during eupnea and during exposure to hypoxia‐hypercapnia, with larger amplitude Pdi observed during tracheal occlusion and in response to bilateral nerve stimulation (Pdi_max_). There was no effect on Pdi amplitude during bilateral phrenic nerve stimulation (Pdi_max_) of very old age (*F*_2,16_ = 0.09, *p* = .92), sex (*F*_2,16_ = 0.35, *p* = .56) or an age × sex interaction (*F*_2,16_ = 1.27, *p* = .31). The mean Pdi_max_ was 70.1 ± 35.3 cm H_2_O at 24 mo, 61.3 ± 23.0 cm H_2_O at 27 mo, and 63.7 ± 38.1 cm H_2_O at 30 mo. The average Pdi_max_ was 65.0 ± 31.7 cm H_2_O, across all age groups (Figure 2).

**Figure 3:**
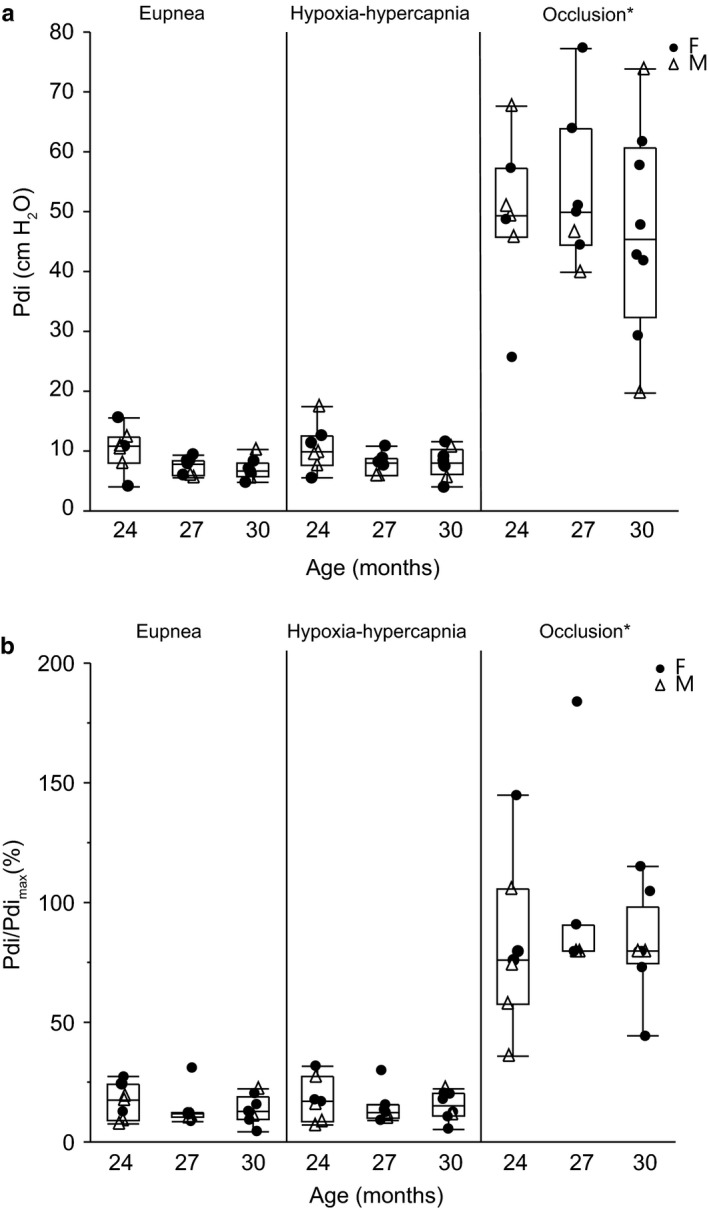
Transdiaphragmatic pressure (Pdi) generation across motor behaviors in 24 (*n* = 7), 27 (*n* = 7), and 30 (*n* = 8) month old female (F; closed circle) and male (M; open triangle) C57BL/6 × 129 mice: eupnea, hypoxia (10% O_2_)‐hypercapnia (5% CO_2_) and tracheal occlusion, presented as absolute values (a), and normalized to Pdi_max_ (b). Data points are the average Pdi amplitude from each animal. Boxplot represents median, 25th and 75th percentile and whiskers 10th and 90th percentiles. Data were analyzed with a mixed linear model with an animal as a random effect (age × behavior × animal). (a) Main effect of behavior (*p* < .01), no effect of age (*p* = .65), or interaction (*p* = .68). (b) Main effect of behavior (*p* < .01), no effect of age (*p* = .64), or interaction (*p* = .77) *significantly higher Pdi generation during tracheal occlusion compared to eupnea and hypoxia‐hypercapnia

The average absolute Pdi amplitude and normalized Pdi amplitude (% absolute Pdi amplitude/ Pdi_max_) across behaviors are shown in Figure 3. There was a main effect of behavior on absolute Pdi (*F*_2,38_ = 212.4, *p* < .01). There was no effect on absolute Pdi amplitude of very old age (*F*_2,19_ = 0.44, *p* = .65) or age × behavior interaction (*F*_4,38_ = 0.59, *p* = .68). The average absolute Pdi was 8.1 ± 2.8 cm H_2_O during eupnea, 8.8 ± 3.0 cm H_2_O during hypoxia‐hypercapnia, and 50.0 ± 14.4 cm H_2_O during tracheal occlusion across all age groups. The absolute Pdi generated during tracheal occlusion was larger than during eupnea and hypoxia‐hypercapnia.

Given the differences across animals in Pdi_max_, Pdi amplitude during each behavior was also normalized to the respective Pdi_max_. There was a main effect of behavior on normalized Pdi (*F*_2,44_ = 158.0, *p* < .01). There was no effect on normalized Pdi amplitude of very old age (*F*_2,22_ = 0.45, *p* = .64) or age × behavior interaction (*F*_4,44_ = 0.46, *p* = .77). The normalized Pdi amplitude was 14.6% ± 4.14% during eupnea, 15.8% ± 4.14% during hypoxia‐hypercapnia, and 86.6% ± 14.5% of Pdi_max_ during tracheal occlusion across all age groups. The normalized Pdi generated during tracheal occlusion was larger than during eupnea and hypoxia‐hypercapnia.

Ventilatory parameters were determined in anesthetized male and female C57BL/6 × 129 mice from Pdi recordings during eupnea and hypoxia‐hypercapnia (Table 3). There was no effect on RR of very old age (*F*_2,38_ = 0.62, *p* = .54), behavior (*F*_1,37_ = 0.23, *p* = .63), or age × behavior interaction (*F*_2,37_ = 0.38, *p* = .68). The RR was 133.7 ± 27.6 min^‐1^ and 130.0 ± 29.3 min^‐1^ during eupnea and hypoxia‐hypercapnia, respectively, across all age groups. There was no effect on duty cycle of very old age (*F*_2,31_ = 1.24, *p* = .30), behavior (*F*_1,31_ = 1.60, *p* = .22), or age × behavior interaction (*F*_2,37_ = 1.04, *p* = .36). The duty cycle was 44.0% ± 7.9% and 42.3% ± 5.9% during eupnea and hypoxia‐hypercapnia, respectively, across all age groups. As an indicator of potential differences in DIAm efficiency, a tension‐time index was also calculated as the Pdi amplitude × duty cycle. There was no effect on tension‐time index of very old age (*F*_2,19_ = 0.18, *p* = .84), behavior (*F*_1,19_ = 2.54, *p* = .13), or age × behavior interaction (*F*_2,19_ = 0.05, *p* = .96). The tension‐time index was 6% ± 3% and 7% ± 3% during eupnea and hypoxia‐hypercapnia, respectively, across all age groups.

**Table 3:** Ventilatory parameters measured in anesthetized C57BL/6 × 129 mice during transdiaphragmatic pressure (Pdi) recordings

Age	Eupnea	Hypoxia‐hypercapnia
Respiratory rate (min^−1^)		
24 mo	135 ± 25	123 ± 24
27 mo	139 ± 27	143 ± 34
30 mo	127 ± 33	125 ± 29
Duty cycle (%)		
24 mo	44 ± 17	38 ± 10
27 mo	45 ± 2	46 ± 2
30 mo	43 ± 5	42 ± 5
Tension‐time index (%)		
24 mo	6.5 ± 3.1	7.2 ± 4.5
27 mo	6.3 ± 3.8	6.7 ± 3.4
30 mo	5.6 ± 2.1	6.2 ± 2.4

Note

Data analyzed by a mixed linear model with animal as a random effect (age × behavior × animal) at 24 (*n* = 7), 27 (*n* = 7) and 30 (*n* = 7) months of age (mo). Respiratory rate: no effect of age (*p* = .62), behavior (*p* = .63), or interaction (*p* = .68). Duty cycle: no effect of age (*p* = .30), behavior (*p* = .22), or interaction (*p* = .36). Tension‐time index: no effect of age (*p* = .84), behavior (*p* = .13), or interaction (*p* = .96). Data shown as mean ± *SD*.

### 3.4 DIAm histo‐morphological analysis

**Figure 4:**
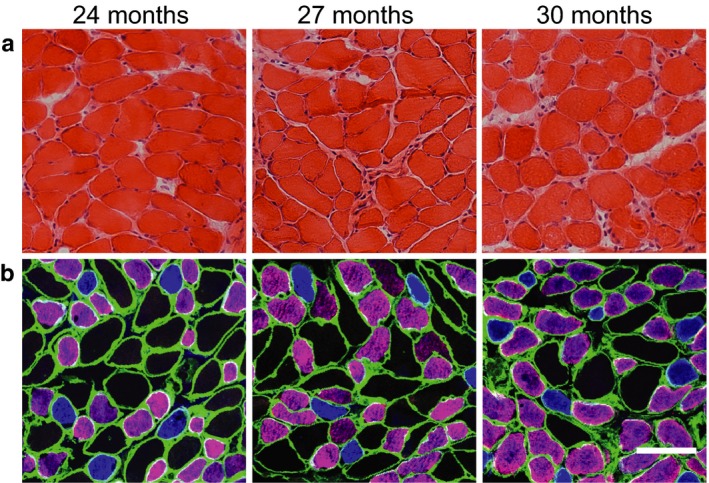
Representative images for histological section of the diaphragm muscle (DIAm) for 24, 27, and 30 month old C57BL/6 × 129 mice: hematoxylin and eosin stain (a); immunohistochemistry based on DIAm myosin heavy chain (MyHC) isoform composition (b): blue—type I fibers, purple—type IIa fibers, and black type IIx and/or IIb fibers. Note lack of evident fiber damage and differences in fiber dimensions across fiber types without obvious changes into very old age. Laminin immunoreactivity is shown in green. Scale bars are 50 µm

The DIAm was collected from 24 mo (*n* = 10), 27 mo (*n* = 5), and 30 mo (*n* = 10) male and female C57BL/6 × 129 mice for measurements of CSA at type‐identified DIAm fibers and proportions of each fiber type. DIAm sections stained with hematoxylin and eosin showed no signs of degenerating fibers, fibrosis, or centrally located nuclei in any of the very old age groups (Figure 4).

**Figure 5:**
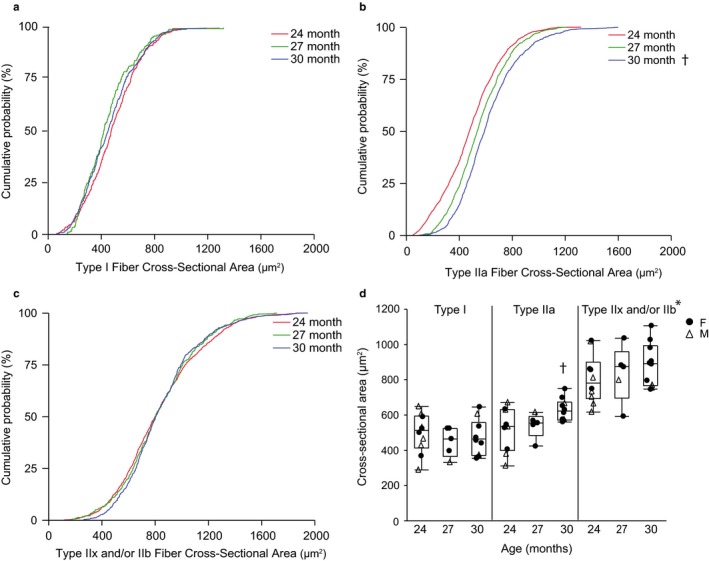
Diaphragm muscle fiber cross‐sectional area at type‐identified fibers in 24 (*n* = 10), 27 (*n* = 5), and 30 (*n* = 10) month old female (F; closed circle) and male (M; open triangle) C57BL/6 × 129 mice. Cumulative distribution function plots for type I fibers (a), type IIa fibers (b), type IIx and/or IIb fibers (c) and average of mean cross‐sectional area for each fiber type per animal (d). Boxplot represents median, 25th and 75th percentile and whiskers 10th and 90th percentiles. Data were analyzed with a mixed linear model with animal as a random effect (age × fiber type × animal). Main effect of fiber type (*p* < .01), age × fiber type interaction (*p* < .01), but no effect of age (*p* = .33). *Type IIx and/or IIb fibers CSA significantly greater than that of type I or IIa fibers across all age groups, †Type IIa CSA significantly greater at 30 mo than 24 mo

Cumulative distribution function plots are shown in Figure 5 to represent the effect of very old age on fiber CSA. There was no effect on CSA of very old age (*F*_2,22_ = 1.14, *p* = .33). There was an effect on CSA of fiber type (*F*_2,7,730_ = 1,283, *p* < .01) and an age × fiber type interaction (*F*_4,7,731_ = 19.8, *p* < .01). Across all age groups, the mean CSA for type IIa fibers (537 ± 227 µm^2^) was less than that of type IIx and/or IIb fibers (844 ± 300 µm^2^), and the mean CSA of type I fibers (472 ± 198 µm^2^) was less than the mean CSA of type IIa and type IIx and/or IIb fibers. There was no effect of age on the CSA of type I or type IIx and/or IIb fibers, but the CSA of type IIa fibers was significantly different between the 24 to 30 mo C57BL/6 × 129 mice.

**Figure 6:**
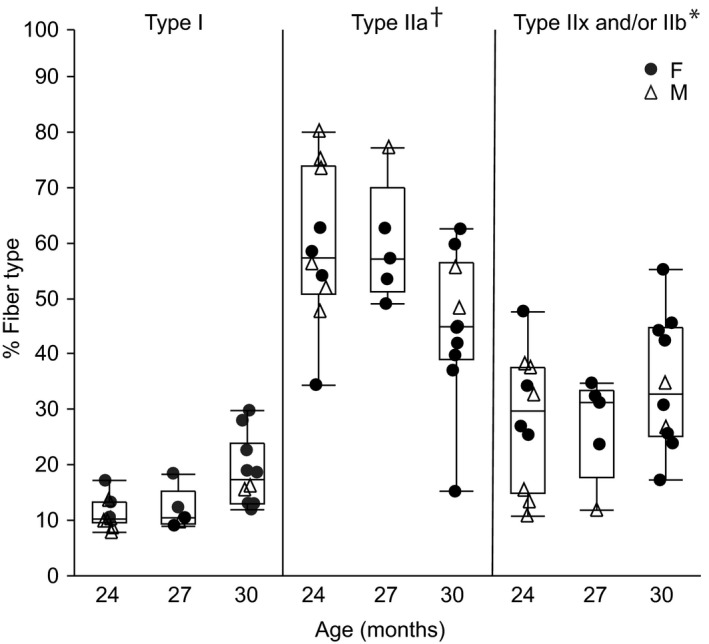
Diaphragm muscle fiber proportions according to fiber type in 24 (*n* = 10), 27 (*n* = 5), and 30 (*n* = 10) month old female (F; closed circle) and male (M; open triangle) C57BL/6 × 129 mice. Data points are average cross‐sectional area from each animal. Boxplot represents median, 25th and 75th percentile and whiskers 10th and 90th percentiles. Data were analyzed with a mixed linear model with animal as a random effect (age × fiber type × animal). Main effect of fiber type (*p* < .01), age × fiber type interaction (*p* < .01), but no effect of age (*p* = 1.00). *Type IIx and/or IIb fiber proportion significantly greater than type I or IIa fibers across all age groups, ^†^Type IIa fiber proportion significantly greater than type I fibers across all age groups

There were relatively minor changes in DIAm fiber type proportions in these very old age groups. On average, a total of ~330 fibers were measured per animal. The average proportion of DIAm muscle fibers of each type at 24, 27, and 30 mo are shown in Figure 6. There was no effect on fiber type proportion of very old age (*F*_2,2_ < 1, *p* = 1.00). There was an effect on fiber type proportion of fiber type (*F*_2,2_ = 89.1, *p* < .01) and an age × fiber type interaction (*F*_4,4_ = 4.34, *p* < .01). From 24 to 30 mo of age, the proportion of type I, type IIa, and type IIx and/or IIb fibers did not change significantly. Overall, the proportion of type I fibers (14%) was less than type IIa fibers (56%) and type IIx and/or IIb fibers (30%). In 24 and 27 mo mice, the proportion of fibers of each type was significantly different in all pairwise comparisons, but in 30 mo mice, the proportion of type IIa fibers and type IIx and/or IIb fibers was no longer significantly different.

The relative contribution by specific fiber type to the total DIAm CSA was calculated based on the CSA of each fiber and the relative proportions per fiber type. There was an effect on the relative contribution of fiber type (*F*_2,2_ = 165.7, *p* < .01), no effect of very old age (*F*_2,2_ < 1, *p* = 1.00), and a fiber type × age interaction (*F*_2,24_ = 2.9, *p* = .03). At 24, 27, and 30 mo, the relative contribution of type I fibers to total DIAm CSA was significantly less than that of type IIa and of type IIx and/or IIb fibers, with contributions of approximately 10% for type I, 45% for type IIa, and 45% for type IIx and/or IIb fibers. In 24 and 27 mo mice, the relative contribution of type IIa and type IIx and/or IIb fibers to total DIAm CSA was not significantly different, but in 30 mo mice, the relative contribution of type IIa fibers to total DIAm CSA compared was significantly greater than that of type IIx and/or IIb fibers (50% vs. 40%, respectively).

## 4 DISCUSSION

The purpose of this study was to examine DIAm sarcopenia into very old age in C57BL/6 × 129 mice during a period (24–30 mo) of substantial reduction in survivorship (~80% vs. ~30%, respectively) (Flurkey, 2009; Turturro et al., 1999). Previous studies in male and female C57BL/6 × 129 mice indicate a ~30% reduction of maximal force generation (Greising, Mantilla, et al., 2013, 2015) and a ~27% decrease in type IIx and/or IIb DIAm fiber CSA in old, 24 mo mice compared to young, 6 mo mice (Greising, Mantilla, et al., 2013). However, there was limited information on DIAm sarcopenia into older age groups. The results of the present study do not support further loss of DIAm function or worsening of sarcopenia into very old age (24 to 30 mo), indicating that survivorship into very old age is predicated by maintenance of a minimal level of force generation by the DIAm. Indeed, there was no effect of very old age on absolute Pdi or normalized Pdi (normalized to Pdi_max_ amplitude) during ventilatory behaviors. Furthermore, there were relatively minor changes in type‐identified DIAm fiber CSA or proportion in male and female C57BL/6 × 129 mice.

### 4.1 Changes in Pdi with survivorship into very old age

Previous studies support the use of Pdi to evaluate force generation by the DIAm and the effects of sarcopenia (Greising, Sieck, et al., 2013; Mantilla et al., 2010; Mantilla & Sieck, 2011b; Medina‐Martinez, Greising, Sieck, & Mantilla, 2015). The American Thoracic Society and the European Respiratory Society Statement on Respiratory Muscle Testing (European & Society, 2002) supports the clinical use of Pdi to study force generation across the full range of motor behaviors accomplished by respiratory muscles such as the DIAm. Ventilatory behaviors can be accomplished across a number of species by generating forces that represent only ~10%–30% of the maximal force generating capacity of the DIAm (Fogarty, Mantilla, & Sieck, 2019; Mantilla et al., 2010; Sieck, 1989). However, higher forces are necessary to accomplish expulsive, airway clearing behaviors such as coughing or sneezing as well as defecation, micturition, and parturition, where co‐activation of the DIAm and abdominal muscles is required (Milano, Grelot, Bianchi, & Iscoe, 1992; Rybak et al., 2008; Shannon, Baekey, Morris, & Lindsey, 1998). Large inspiratory efforts contribute to the generation of higher abdominal pressures which subsequently enable the increased intra‐thoracic pressure necessary for airway clearance (Fogarty & Sieck, 2019; Mantilla & Sieck, 2013). In the present study, the average absolute Pdi across all age groups was 8.1 ± 2.8 cm H_2_O during eupnea, 8.8 ± 2.9 cm H_2_O during hypoxia‐hypercapnia, and 50.0 ± 14.4 cm H_2_O during tracheal occlusion. The Pdi measured across all behaviors in 24 mo mice was consistent with our previous findings which compared 6 and 24 mo C57BL/6 × 129 mice and documented a ~30% decrease in Pdi_max_ (Greising, Mantilla, et al., 2015). Of note, in the present study from 24 to 30 mo of age, the absolute and normalized Pdi and ventilatory parameters (RR, duty cycle, tension‐time index) did not change during ventilatory behaviors (eupnea or hypoxia‐hypercapnia). In all age groups (24, 27, and 30 mo), Pdi during tracheal occlusion was significantly higher than during eupnea or hypoxia‐hypercapnia. Between the ages of 6 to 24 mo in mice, we previously reported that there is a ~40% decrease in maximal Pdi generation during tracheal occlusion, reflective of higher forces necessary for this behavior (Greising, Mantilla, et al., 2015). Decreased maximal force can impair non‐ventilatory behaviors like the larynges coughing reflex, Valsalva maneuver, and vomiting, all of which require large, positive intra‐abdominal pressures (Fogarty & Sieck, 2019). However, in the present study, it is likely that there will not be a further functional impact on DIAm force generation from 24 to 30 mo of age, as Pdi remained consistent within each behavior (eupnea, hypoxia‐hypercapnia, and tracheal occlusion). Although measurements of DIAm mass, including non‐contractile mass (e.g., collagen), are necessary to evaluate changes in specific force and thus the mechanism of aging effects on muscle mass and force (i.e., Pdi) (Clark & Manini, 2008; Gosselin, Martinez, Vailas, & Sieck, 1993), our previous reports do not support significant changes in the contribution of non‐contractile elements to DIAm mass in 24 mo C57BL/6 × 129 male and female mice (Greising, Mantilla, et al., 2013). Specifically, the fraction of total muscle CSA comprised by interstitial space displayed only a very slight increase in 24 mo C57BL/6 × 129 mice compared to 6 mo mice, and the fraction of contractile proteins myosin and actin (expressed per total protein) decreased minimally in this same period.

The changes in respiratory mechanics associated with old age are well established. With very old age, there is an increase in functional reserve capacity and in alveolar size (Huang, Rabold, Schofield, Mitzner, & Tankersley, 2007; Massa, Groves, Jaggernauth, Laskin, & Gow, 2017). In addition, respiratory system resistance changes minimally from 6 to 18 mo (~1%–2% decrease per month), and increases more rapidly from 24 to 30 mo, ~5% per month (Elliott, Mantilla, et al., 2016). The changes associated with respiratory mechanics may compound age‐related effects including contributing to increased difficulty with airway clearance. The cumulative effects of the changes in respiratory system mechanics and limited DIAm force generation may lead to further deterioration in the force generating capacity of the DIAm, which would compromise survivorship. In the present study, Pdi_max_ does not change, reflecting a maintained force generating capacity into very old age, and therefore, changes in respiratory mechanics (alveolar space, residual volume, functional residual capacity, and chest compliance) likely play a limited role in surviving mice (Elliott, Mantilla, et al., 2016; Khurram et al., 2018).

The results of the present study show an absence of evidence for sex‐related differences in Pdi at 24, 27, and 30 mo in C57BL/6 × 129 mice. This is consistent with a previous study in C57BL/6 × 129 mice that found an age‐related decline in Pdi from 6 to 24 mo that is not different between sexes (Greising, Mantilla, et al., 2015). Similarly, Fisher344 rats display an age‐related decline in Pdi from 6 to 24 mo that is not different between sexes (Khurram et al., 2018). Collectively, sex does not appear to be an influencing factor in relation to DIAm function in old age.

### 4.2 Diaphragm muscle fiber changes with survivorship into very old age

In several previous reports, we documented fiber type selective atrophy of type IIx and/or IIb DIAm fibers in old, 24 mo mice compared to young, 6 mo C57BL/6 × 129 mice (Greising, Medina‐Martínez, et al., 2015; Greising, Sieck, et al., 2013; Greising, Stowe, et al., 2015). The CSA measurements at type‐identified DIAm fibers for 24 mo mice in the present study are in agreement with these previous reports (Greising, Medina‐Martínez, et al., 2015; Greising, Sieck, et al., 2013; Greising, Stowe, et al., 2015). In the present study, DIAm CSA for type I or type IIx and/or IIb fibers did not change from 24 to 30 mo of age. However, there was a ~30% increase in the CSA of type IIa fibers from 24 to 30 mo. Also, consistent with our previous studies, the proportion of muscle fibers in the mouse DIAm of each type is ~15% for type I, ~55% for type IIa and ~30% for type IIx and/or IIb fibers. Between 6 and 24 mo, we previously reported an increase in the proportion of type IIa fibers and a decrease in the proportion of type IIx and/or IIb fibers in C57BL/6 × 129 mice (Greising, Sieck, et al., 2013; Greising, Stowe, et al., 2015). The results of the present study are generally consistent with these previous findings in 24 mo mice, and into very old age, the proportions of type‐identified DIAm fibers did not change further. The relative contribution of type IIa fibers to total DIAm CSA increased between 6 and 24 mo in C57BL/6 × 129 mice (Greising, Sieck, et al., 2013), and increased further to 30 mo (present study). It is possible that the increase in type IIa DIAm fiber CSA (into very old age) and the increase in type IIa proportions and relative contribution to total CSA reflect hybrid fibers with co‐expression of MyHC_2A_, and MyHC_2X_ and MyHC_2B_ isoforms as a result of denervation of skeletal muscle fibers and re‐innervation through axonal sprouting to neighboring fibers (Greising, Medina‐Martínez, et al., 2015).

### 4.3 Mechanisms of neuromuscular dysfunction in old age

Denervation may result from neuromuscular dysfunction, trauma, or disease (Greising, Sieck, et al., 2013; Mantilla & Sieck, 2009; Rowan et al., 2012), as well as old age. With age, there is an increased presence of hybrid fibers with evidence of co‐expression of MyHC_2A_ (type IIa fibers) with both MyHC_Slow_ (type I fibers) and MyHC_2X_ or MyHC_2B_ (type IIx and/or IIb fibers) (Zhan, Miyata, Prakash, & Sieck, 1997). Therefore, it is likely that there is co‐expression of MyHC_2A_, and MyHC_2X_ and MyHC_2B_ isoforms in these hybrid DIAm fibers. As in previous studies, muscle fiber types were classified based on MyHC expression (Greising, Mantilla, et al., 2013; Mantilla & Sieck, 2011b; Sieck, 1991, 1994), with type I and type IIa fibers directly identified based on MyHC_slow_ and MyHC_2A_ isoform immunoreactivity. The absence of immunoreactivity was taken to indicate type IIx and/or IIb fibers. However, due to unavailable and unreliable labeling techniques, staining for fiber type co‐expression was not further examined (Greising, Medina‐Martínez, et al., 2015).

Previous studies have provided qualitative and quantitative evidence of skeletal muscle clustering as a response to muscle fiber denervation (Karpati & Engel, 1968; Kugelberg, 1976; Vleggeert‐Lankamp et al., 2005) and aging (Greising, Medina‐Martínez, et al., 2015). Indeed, the inter‐fiber distance between type IIx and/or IIb fibers in the DIAm increases from 6 to 24 mo in C57BL/6 × 129 mice consistent with clustering of other fiber types, primarily type IIa fibers. Furthermore, there is an increase in type IIa fiber proportion and a decrease in type IIx and/ or IIb fiber proportion from 6 to 24 mo in C57BL/6 × 129 mice (Greising, Medina‐Martínez, et al., 2015). It is likely that the increase in inter‐fiber distance of type IIx and/or IIb and the change in fiber type proportions is reflective of denervation of type IIx and/or IIb fibers and re‐innervation of the most abundant type IIa fibers (Greising, Medina‐Martínez, et al., 2015).

### 4.4 Clinical implications

The heterogeneity of the DIAm motor unit and muscle fiber contractile and fatigue properties determines the range of motor behaviors accomplished by the DIAm and shows matched fatigue properties to functional demands across various species (Greising, Mantilla, et al., 2015; Sieck & Fournier, 1989; Sieck et al., 1989). Assuming an orderly recruitment of motor units, slow‐twitch type S units and fast‐twitch fatigue resistant (type FR) units are recruited before fast‐twitch fatigue intermediate (type FInt) and fatigable (type FF) units (Fogarty, Mantilla, et al., 2019; Mantilla & Sieck, 2011b). In the DIAm, ventilatory behaviors, including breathing, are reflective of recruitment of type I fibers (type S motor units) and type IIa fibers (type FR motor units), and higher force, non‐ventilatory behaviors reflect recruitment of type IIx and/or IIb fibers (type FInt and FF motor units) (Mantilla et al., 2010; Mantilla & Sieck, 2011b). Selective atrophy of type IIx and/or IIb fibers may dictate a decreased ability to perform non‐ventilatory behaviors such as coughing and sneezing in old age (Greising, Mantilla, et al., 2013; Greising, Medina‐Martínez, et al., 2015; Greising, Stowe, et al., 2015), which thus may ultimately determine survivorship.

DIAm changes associated with sarcopenia are likely implicated in a variety of pathologies including pneumonia and respiratory distress syndrome (Greising, Mantilla, et al., 2013, 2015). An underlying factor affecting these diseases is due to the fiber type‐selective atrophy of type IIx and/or IIb fibers, which diminishes the body's capacity to perform expulsive behaviors necessary for airway clearance. In addition to impaired respiratory function, older populations also have increased adverse outcomes that coincide with increasing age, such as higher risks of falls and fractures, frailty, and mortality (Beaudart et al., 2016; Landers, Hunter, Wetzstein, Bamman, & Weinsier, 2001; Lauretani et al., 2003; Rizzoli et al., 2013). Further study is necessary to compare and contrast aging effects on locomotor and respiratory systems.

## 5 CONCLUSIONS

The present study does not support further loss of DIAm function or worsening of sarcopenia into very old age (24 to 30 mo), indicating that survivorship is predicated by maintenance of a minimal level of force generation by the DIAm. Sarcopenia of the respiratory muscles will impact both ventilatory and non‐ventilatory behaviors (Khurram et al., 2018; Mantilla, Seven, & Sieck, 2014; Mantilla & Sieck, 2011b; Sieck, 1988, 1989; Sieck & Fournier, 1989), and from 6 to 24 mo there is evidence of sarcopenia that is maintained into very old age (Greising, Mantilla, et al., 2013, 2015; Greising, Medina‐Martínez, et al., 2015; Greising, Stowe, et al., 2015). Sarcopenia in the DIAm is likely reflective of denervation and re‐innervation of neighboring muscle fibers as a response to disease or injury. The CSA and proportion of type IIx and/or IIb fibers and Pdi across all ventilatory parameters were not affected by survivorship into very old age. Collectively, these results may suggest a minimal level of muscle function is required to sustain respiration and to be compatible with life.

## CONFLICT OF INTEREST

The authors declared no conflicts of interest, financial or other.

## AUTHOR CONTRIBUTIONS

PV, HMG, GCS, and CBM contributed to experimental design and conceptual framework. PV, AKV, WZZ, HMG, GCS, and CBM contributed to data collection and analysis. PV, AKV, HMG, GCS, and CBM involved in drafting article. Final submission has been read and approved by all authors.

## References

[phy214305-bib-0001] Bazzy, A. R. , & Haddad, G. G. (1984). Diaphragmatic fatigue in unanesthetized adult sheep. Journal of Applied Physiology, 57(1), 182–190. 6469779 10.1152/jappl.1984.57.1.182

[phy214305-bib-0002] Beaudart, C. , McCloskey, E. , Bruyère, O. , Cesari, M. , Rolland, Y. , Rizzoli, R. , … Cooper, C. . (2016). Sarcopenia in daily practice: Assessment and management. BMC Geriatrics, 16, 170. 27716195 10.1186/s12877-016-0349-4 PMC5052976

[phy214305-bib-0003] Bellemare, F. , & Grassino, A. (1982). Effect of pressure and timing of contraction on human diaphragm fatigue assessed by phrenic nerve stimulation. Journal of Applied Physiology, 53, 1190–1195. 7174413 10.1152/jappl.1982.53.5.1190

[phy214305-bib-0004] Cesari, M. , Fielding, R. A. , Pahor, M. , Goodpaster, B. , Hellerstein, M. , van Kan, G. A. , … Vellas, B. (2012). Biomarkers of sarcopenia in clinical trials‐recommendations from the International Working Group on Sarcopenia. J Cachexia Sarcopenia Muscle, 3, 181–190. 22865205 10.1007/s13539-012-0078-2 PMC3424187

[phy214305-bib-0005] Clark, B. C. , & Manini, T. M. (2008). Sarcopenia =/= dynapenia. Journals of Gerontology. Series A, Biological Sciences and Medical Sciences, 63, 829–834. 10.1093/gerona/63.8.829 18772470

[phy214305-bib-0006] Elliott, J. E. , Greising, S. M. , Mantilla, C. B. , & Sieck, G. C. (2016). Functional impact of sarcopenia in respiratory muscles. Respiratory Physiology & Neurobiology, 226, 137–146. 26467183 10.1016/j.resp.2015.10.001 PMC4838572

[phy214305-bib-0007] Elliott, J. E. , Mantilla, C. B. , Pabelick, C. M. , Roden, A. C. , & Sieck, G. C. (2016). Aging‐related changes in respiratory system mechanics and morphometry in mice. American Journal of Physiology. Lung Cellular and Molecular Physiology, 311, L167–L176. 27288490 10.1152/ajplung.00232.2016 PMC4967189

[phy214305-bib-0008] Elliott, J. E. , Omar, T. S. , Mantilla, C. B. , & Sieck, G. C. (2016). Diaphragm muscle sarcopenia in Fischer 344 and Brown Norway rats. Experimental Physiology, 101, 883–894. 27126607 10.1113/EP085703 PMC4930373

[phy214305-bib-0009] Enad, J. G. , Fournier, M. , & Sieck, G. C. (1989). Oxidative capacity and capillary density of diaphragm motor units. Journal of Applied Physiology, 67, 620–627. 2529236 10.1152/jappl.1989.67.2.620

[phy214305-bib-0010] European, R. S. & Society, A. T (2002). ATS/ERS Statement on respiratory muscle testing. American Journal of Respiratory and Critical Care Medicine, 166, 518. 12186831 10.1164/rccm.166.4.518

[phy214305-bib-0011] Fielding, R. A. , Vellas, B. , Evans, W. J. , Bhasin, S. , Morley, J. E. , Newman, A. B. , … Zamboni, M. . (2011). Sarcopenia: An undiagnosed condition in older adults. Current consensus definition: Prevalence, etiology, and consequences. International working group on sarcopenia. Journal of the American Medical Directors Association, 12, 249–256. 21527165 10.1016/j.jamda.2011.01.003 PMC3377163

[phy214305-bib-0012] Flurkey K. (Ed.) (2009). The Jackson laboratory handbook on genetically standardized mice. ME: Bar Harbor.

[phy214305-bib-0013] Fogarty, M. J. , Gonzalez Porras, M. A. , Mantilla, C. B. , & Sieck, G. C. (2019). Diaphragm neuromuscular transmission failure in aged rats. Journal of Neurophysiology, 122, 93–104. 31042426 10.1152/jn.00061.2019 PMC6689786

[phy214305-bib-0014] Fogarty, M. J. , Mantilla, C. B. , & Sieck, G. C. (2019). Impact of sarcopenia on diaphragm muscle fatigue. Experimental Physiology, 104, 1090–1099. 30924589 10.1113/EP087558 PMC6602803

[phy214305-bib-0015] Fogarty, M. J. , & Sieck, G. C. (2019). Evolution and functional differentiation of the diaphragm muscle of mammals. Comprehensive Physiology, 9, 715–766. 30873594 10.1002/cphy.c180012 PMC7082849

[phy214305-bib-0016] Fournier, M. , & Sieck, G. C. (1988). Mechanical properties of muscle units in the cat diaphragm. Journal of Neurophysiology, 59, 1055–1066. 3367195 10.1152/jn.1988.59.3.1055

[phy214305-bib-0017] Gill, L. C. , Mantilla, C. B. , & Sieck, G. C. (2015). Impact of unilateral denervation on transdiaphragmatic pressure. Respiratory Physiology & Neurobiology, 210, 14–21. 25641347 10.1016/j.resp.2015.01.013 PMC4449269

[phy214305-bib-0018] Gonzalez Porras, M. A. , Sieck, G. C. , & Mantilla, C. B. (2018). Impaired autophagy in motor neurons: A final common mechanism of injury and death. Physiology, 33, 211–224. 29638184 10.1152/physiol.00008.2018 PMC5966659

[phy214305-bib-0019] Gosselin, L. E. , Martinez, D. A. , Vailas, A. C. , & Sieck, G. C. (1993). Interstitial space and collagen alterations of the developing rat diaphragm. Journal of Applied Physiology, 74, 2450–2455. 7687597 10.1152/jappl.1993.74.5.2450

[phy214305-bib-0020] Greising, S. M. , Mantilla, C. B. , Gorman, B. A. , Ermilov, L. G. , & Sieck, G. C. (2013). Diaphragm muscle sarcopenia in aging mice. Experimental Gerontology, 48, 881–887. 23792145 10.1016/j.exger.2013.06.001 PMC3750110

[phy214305-bib-0021] Greising, S. M. , Mantilla, C. B. , Medina‐Martinez, J. S. , Stowe, J. M. , & Sieck, G. C. (2015). Functional impact of diaphragm muscle sarcopenia in both male and female mice. American Journal of Physiology. Lung Cellular and Molecular Physiology, 309, L46–L52. 25934669 10.1152/ajplung.00064.2015 PMC4491513

[phy214305-bib-0022] Greising, S. M. , Mantilla, C. B. , & Sieck, G. C. (2016). Functional measurement of respiratory muscle motor behaviors using transdiaphragmatic pressure. Methods in Molecular Biology, 1460, 309–319. 27492181 10.1007/978-1-4939-3810-0_21 PMC5562284

[phy214305-bib-0023] Greising, S. M. , Medina‐Martínez, J. S. , Vasdev, A. K. , Sieck, G. C. , & Mantilla, C. B. (2015). Analysis of muscle fiber clustering in the diaphragm muscle of sarcopenic mice. Muscle and Nerve, 52, 76–82. 25808550 10.1002/mus.24641 PMC4474759

[phy214305-bib-0024] Greising, S. M. , Sieck, D. C. , Sieck, G. C. , & Mantilla, C. B. (2013). Novel method for transdiaphragmatic pressure measurements in mice. Respiratory Physiology & Neurobiology, 188, 56–59. 23632282 10.1016/j.resp.2013.04.018 PMC3699961

[phy214305-bib-0025] Greising, S. M. , Stowe, J. M. , Sieck, G. C. , & Mantilla, C. B. (2015). Role of TrkB kinase activity in aging diaphragm neuromuscular junctions. Experimental Gerontology, 72, 184–191. 26517952 10.1016/j.exger.2015.10.013 PMC4667365

[phy214305-bib-0026] Greising, S. M. , Vasdev, A. K. , Zhan, W. Z. , Sieck, G. C. , & Mantilla, C. B. (2017). Chronic TrkB agonist treatment in old age does not mitigate diaphragm neuromuscular dysfunction. Physiological Reports, 5, e13103. 28082429 10.14814/phy2.13103 PMC5256161

[phy214305-bib-0027] Heron, M. (2017). Deaths: Leading causes for 2015. National Vital Statistics Reports, 66, 1–76. 29235984

[phy214305-bib-0028] Holter, P. H. , Kierulf, P. , & Refsum, H. E. (1987). Haemoglobin O2 binding in newborn and adult rabbits. Acta Physiologica Scandinavica, 130, 349–356. 3111176 10.1111/j.1748-1716.1987.tb08147.x

[phy214305-bib-0029] Huang, K. , Rabold, R. , Schofield, B. , Mitzner, W. , & Tankersley, C. G. (2007). Age‐dependent changes of airway and lung parenchyma in C57BL/6J mice. Journal of Applied Physiology, 102, 200–206. 16946023 10.1152/japplphysiol.00400.2006

[phy214305-bib-0030] Karpati, G. , & Engel, W. K. (1968). Correlative histochemical study of skeletal muscle after suprasegmental denervation, peripheral nerve section, and skeletal fixation. Neurology, 18, 681–692. 4233750 10.1212/wnl.18.7.681

[phy214305-bib-0031] Khurram, O. U. , Fogarty, M. J. , Sarrafian, T. L. , Bhatt, A. , Mantilla, C. B. , & Sieck, G. C. (2018). Impact of aging on diaphragm muscle function in male and female Fischer 344 rats. Physiological Reports, 6, e13786. 29981218 10.14814/phy2.13786 PMC6035336

[phy214305-bib-0032] Khurram, O. U. , Sieck, G. C. , & Mantilla, C. B. (2017). Compensatory effects following unilateral diaphragm paralysis. Respiratory Physiology & Neurobiology, 246, 39–46. 28790008 10.1016/j.resp.2017.07.007 PMC5624837

[phy214305-bib-0033] Kugelberg, E. (1976). The motor unit: Anatomy and histochemical functional correlations. Rivista di Patologia Nervosa e Mentale, 97, 251–258. 146250

[phy214305-bib-0034] Landers, K. A. , Hunter, G. R. , Wetzstein, C. J. , Bamman, M. M. , & Weinsier, R. L. (2001). The interrelationship among muscle mass, strength, and the ability to perform physical tasks of daily living in younger and older women. Journals of Gerontology. Series A, Biological Sciences and Medical Sciences, 56, B443–B448. 10.1093/gerona/56.10.b443 11584029

[phy214305-bib-0035] Lauretani, F. , Russo, C. R. , Bandinelli, S. , Bartali, B. , Cavazzini, C. , di Iorio, A. , … Ferrucci, L. (2003). Age‐associated changes in skeletal muscles and their effect on mobility: An operational diagnosis of sarcopenia. Journal of Applied Physiology, 1985(95), 1851–1860. 10.1152/japplphysiol.00246.2003 14555665

[phy214305-bib-0036] Mantilla, C. B. , Seven, Y. B. , & Sieck, G. C. (2014). Convergence of pattern generator outputs on a common mechanism of diaphragm motor unit recruitment. Progress in Brain Research, 209, 309–329. 24746055 10.1016/B978-0-444-63274-6.00016-3 PMC4154308

[phy214305-bib-0037] Mantilla, C. B. , Seven, Y. B. , Zhan, W. Z. , & Sieck, G. C. (2010). Diaphragm motor unit recruitment in rats. Respiratory Physiology & Neurobiology, 173, 101–106. 20620243 10.1016/j.resp.2010.07.001 PMC2919593

[phy214305-bib-0038] Mantilla, C. B. , & Sieck, G. C. (2009). Neuromuscular adaptations to respiratory muscle inactivity. Respiratory Physiology & Neurobiology, 169, 133–140. 19744580 10.1016/j.resp.2009.09.002 PMC2783688

[phy214305-bib-0039] Mantilla, C. B. , & Sieck, G. C. (2011a). Age‐related remodeling of neuromuscular junctions InLynch G. S., (Ed.), Sarcopenia ‐ age‐related muscle wasting and weakness. Netherlands: Springer.

[phy214305-bib-0040] Mantilla, C. B. , & Sieck, G. C. (2011b). Phrenic motor unit recruitment during ventilatory and non‐ventilatory behaviors. Respiratory Physiology & Neurobiology, 179, 57–63. 21763470 10.1016/j.resp.2011.06.028 PMC3183333

[phy214305-bib-0041] Mantilla, C. B. , & Sieck, G. C. (2013). Impact of diaphragm muscle fiber atrophy on neuromotor control. Respiratory Physiology & Neurobiology, 189, 411–418. 23831121 10.1016/j.resp.2013.06.025 PMC3829686

[phy214305-bib-0042] Massa, C. B. , Groves, A. M. , Jaggernauth, S. U. , Laskin, D. L. , & Gow, A. J. (2017). Histologic and biochemical alterations predict pulmonary mechanical dysfunction in aging mice with chronic lung inflammation. PLoS Computational Biology, 13, e1005570. 28837561 10.1371/journal.pcbi.1005570 PMC5570219

[phy214305-bib-0043] Medina‐Martinez, J. S. , Greising, S. M. , Sieck, G. C. , & Mantilla, C. B. (2015). Semi‐automated assessment of transdiaphragmatic pressure variability across motor behaviors. Respiratory Physiology & Neurobiology, 215, 73–81. 26003850 10.1016/j.resp.2015.05.009 PMC4490018

[phy214305-bib-0044] Messa, G. A. M. , Piasecki, M. , Hill, C. , McPhee, J. S. , Tallis, J. , & Degens, H. (2019). Morphological alterations of mouse skeletal muscles during early ageing are muscle specific. Experimental Gerontology, 125, 110684. 31400439 10.1016/j.exger.2019.110684

[phy214305-bib-0045] Milano, S. , Grelot, L. , Bianchi, A. L. , & Iscoe, S. (1992). Discharge patterns of phrenic motoneurons during fictive coughing and vomiting in decerebrate cats. Journal of Applied Physiology, 73, 1626–1636. 1447114 10.1152/jappl.1992.73.4.1626

[phy214305-bib-0046] Miller, R. A. , & Nadon, N. L. (2000). Principles of animal use for gerontological research. Journals of Gerontology. Series A, Biological Sciences and Medical Sciences, 55, B117–B123. 10.1093/gerona/55.3.B117 PMC7110343 10795715

[phy214305-bib-0047] Navarro, A. , Sanchez Del Pino, M. J. , Gomez, C. , Peralta, J. L. , & Boveris, A. (2002). Behavioral dysfunction, brain oxidative stress, and impaired mitochondrial electron transfer in aging mice. American Journal of Physiology: Regulatory, Integrative and Comparative Physiology, 282, R985–R992. 10.1152/ajpregu.00537.2001 11893601

[phy214305-bib-0048] Rizzoli, R. , Reginster, J. Y. , Arnal, J. F. , Bautmans, I. , Beaudart, C. , Bischoff‐Ferrari, H. , … Bruyere, O. (2013). Quality of life in sarcopenia and frailty. Calcified Tissue International, 93, 101–120. 23828275 10.1007/s00223-013-9758-y PMC3747610

[phy214305-bib-0049] Rowan, S. L. , Rygiel, K. , Purves‐Smith, F. M. , Solbak, N. M. , Turnbull, D. M. , & Hepple, R. T. (2012). Denervation causes fiber atrophy and myosin heavy chain co‐expression in senescent skeletal muscle. PLoS ONE, 7, e29082. 22235261 10.1371/journal.pone.0029082 PMC3250397

[phy214305-bib-0050] Rybak, I. A. , O'Connor, R. , Ross, A. , Shevtsova, N. A. , Nuding, S. C. , Segers, L. S. , … Lindsey, B. G. (2008). Reconfiguration of the pontomedullary respiratory network: A computational modeling study with coordinated in vivo experiments. Journal of Neurophysiology, 100, 1770–1799. 10.1152/jn.90416.2008 18650310 PMC2576193

[phy214305-bib-0051] Shannon, R. , Baekey, D. M. , Morris, K. F. , & Lindsey, B. G. (1998). Ventrolateral medullary respiratory network and a model of cough motor pattern generation. Journal of Applied Physiology, 84, 2020–2035. 9609797 10.1152/jappl.1998.84.6.2020

[phy214305-bib-0052] Sieck, G. C. (1988). Diaphragm muscle: Structural and functional organization. Clinics in Chest Medicine, 9, 195–210. 3292123

[phy214305-bib-0053] Sieck, G. C. (1989). Recruitment and frequency coding of diaphragm motor units during ventilatory and non‐ventilatory behaviors In Swanson G. D., Grodins F. S., & Hughson R. L. (Eds.), Respiratory control. New York: Plenum Press.

[phy214305-bib-0054] Sieck, G. C. (1991). Neural control of the inspiratory pump. NIPS, 6, 260–264.

[phy214305-bib-0055] Sieck, G. C. (1994). Physiological effects of diaphragm muscle denervation and disuse. Clinics in Chest Medicine, 15, 641–659. 7867280

[phy214305-bib-0056] Sieck, G. C. , & Fournier, M. (1989). Diaphragm motor unit recruitment during ventilatory and nonventilatory behaviors. Journal of Applied Physiology, 66, 2539–2545. 2745316 10.1152/jappl.1989.66.6.2539

[phy214305-bib-0057] Sieck, G. C. , Fournier, M. , & Enad, J. G. (1989). Fiber type composition of muscle units in the cat diaphragm. Neuroscience Letters, 97, 29–34. 2521928 10.1016/0304-3940(89)90134-1

[phy214305-bib-0058] Turturro, A. , Witt, W. W. , Lewis, S. , Hass, B. S. , Lipman, R. D. , & Hart, R. W. (1999). Growth curves and survival characteristics of the animals used in the Biomarkers of Aging Program. Journals of Gerontology. Series A, Biological Sciences and Medical Sciences, 54, B492–501. 10.1093/gerona/54.11.b492 10619312

[phy214305-bib-0059] Vleggeert‐Lankamp, C. L. , de Ruiter, G. C. , Wolfs, J. F. , Pego, A. P. , Feirabend, H. K. , Lakke, E. A. , & Malessy, M. J. (2005). Type grouping in skeletal muscles after experimental reinnervation: Another explanation. European Journal of Neuroscience, 21, 1249–1256. 15813934 10.1111/j.1460-9568.2005.03954.x

[phy214305-bib-0060] Yang, M. , Hu, X. , Wang, H. , Zhang, L. , Hao, Q. , & Dong, B. (2017). Sarcopenia predicts readmission and mortality in elderly patients in acute care wards: A prospective study. J Cachexia Sarcopenia Muscle, 8, 251–258. 27896949 10.1002/jcsm.12163 PMC5377397

[phy214305-bib-0061] Yuan, R. , Tsaih, S. W. , Petkova, S. B. , Marin De Evsikova, C. , Xing, S. , Marion, M. A. , … Paigen, B . (2009). Aging in inbred strains of mice: Study design and interim report on median lifespans and circulating IGF1 levels. Aging Cell, 8, 277–287. 19627267 10.1111/j.1474-9726.2009.00478.x PMC2768517

[phy214305-bib-0062] Zhan, W. Z. , Miyata, H. , Prakash, Y. S. , & Sieck, G. C. (1997). Metabolic and phenotypic adaptations of diaphragm muscle fibers with inactivation. Journal of Applied Physiology, 82, 1145–1153. 9104851 10.1152/jappl.1997.82.4.1145

